# Experimental and Numerical Investigation of End-Tab Geometry Effects on the Compressive Response of Ultra-Thick CFRP Laminates

**DOI:** 10.3390/polym18192342

**Published:** 2026-09-25

**Authors:** Yue Chen, Xu Liang, Liqun Tian, Hao Zhang, Baoxuan Wang, Jian Wang, Titao Wang

**Affiliations:** 1 Ocean College, Zhejiang University, Zhoushan 316021, China; chen-yue@zju.edu.cn (Y.C.);; 2 Shanghai Marine Equipment Research Institute, Shanghai 200031, China; 3 Donghai Laboratory, Zhoushan 316021, China

**Keywords:** ultra-thick laminate, carbon-fibre-reinforced polymer, compression testing, end-tab taper, load transfer, failure initiation

## Abstract

Compression testing of ultra-thick carbon-fibre-reinforced polymer (CFRP) laminates requires high loads to be transferred over a limited gripping length, while the stiffness discontinuity at the end-tab termination can initiate unintended local failure. This study examines the effects of taper geometry and tab thickness through combined-loading compression tests on four end-tab configurations, surface-strain measurements, fractography, and three-dimensional finite-element analysis. Multiple intralaminar failure indices were evaluated, and a Ye-type index was used to assess interlaminar initiation risk at the free edge. For the 20 mm thick tabs, introducing a 25 mm taper increased the mean compressive strength by 9.76%. At comparable nominal stresses near 330 MPa, the representative abrupt-ended specimen exhibited approximately twice the strain ranges in the shear and through-thickness directions of the representative tapered specimen. The numerical results showed that the tab-end stiffness gradient governed interlaminar load transfer: increasing taper length reduced the axial-stress gradient and the local interlaminar shear and through-thickness stress concentrations, with the calculated peak S13 decreasing from 48.01 to 18.36 MPa, while shifting the governing Hashin initiation mode from matrix compression to fibre compression. The measured strain localization and the observed fibre compression–shear fracture, matrix shear cracking, and delamination were qualitatively consistent with the numerically identified three-dimensional stress concentrations. These results demonstrate that end-tab transition geometry governs local failure initiation and the measured load-carrying capacity of ultra-thick CFRP compression specimens.

## 1. Introduction

Compression failure in CFRP laminates results from interactions among initial fibre misalignment, nonlinear matrix shear deformation, fibre kinking, interfacial cracking, and interlaminar separation within a confined region [1,2,3,4,5,6]. These interacting mechanisms make the measured compressive response sensitive to geometric imperfections, end constraints, and the method of load introduction. For laminates with thicknesses of several tens of millimetres, the required test load increases substantially with laminate thickness and specimen width. This challenge becomes particularly pronounced in the approximately 30 mm thickness regime examined here, which is referred to as ultra-thick in related studies [7,8]. At this scale, the specimens behave increasingly as structural-scale components: free-edge stresses, interply deformation compatibility, and end-load transfer can alter failure initiation and apparent strength, while three-dimensional and interlaminar mechanisms become more prominent.

Within this regime, the measured compression response is also sensitive to laminate and defect-scale features. Previous studies have shown that thickness, local wrinkles, delamination, and ply-free edges can alter compressive strength and macroscopic fracture morphology [8,9,10,11,12,13]. Gao et al. showed through experiments and three-dimensional simulations of ultra-thick CFRP laminates that intralaminar failure and interlaminar damage require separate assessment and that conventional failure criteria respond differently to local three-dimensional stress states [8]. Reliable testing therefore requires a specimen and tab arrangement that introduces load into the gauge section without end crushing or premature failure at the tab termination [14,15]. Related studies have further shown that the matrix- and interface-dominated response of carbon- and glass-fibre composite systems can be altered by the test setup [14,15].

Composite compression tests commonly use shear loading, end loading, or a combination of the two [16,17]. Combined loading distributes the transfer demand between bearing at the specimen ends and shear along the tab surfaces, but specimen proportions in established standards were developed mainly for thin or moderately thick laminates. End tabs protect the gripped ends and increase the load-transfer area; however, their termination introduces a transition from the combined CFRP-tab section to the untabbed CFRP section. An abrupt tab end concentrates this stiffness change over a short distance and can generate high interlaminar shear and through-thickness stresses, whereas a tapered end provides a gradual cross-sectional transition and extends the distance over which the load is redistributed. Although loading method, laminate thickness, and tab configuration are known to affect measured compressive properties [18,19,20,21], the effects of taper length and tab thickness on load transfer and failure initiation in CFRP compression specimens approximately 30 mm thick remain insufficiently quantified.

Numerical assessment also depends on the selected failure criterion. Maximum-stress and maximum-strain criteria evaluate individual stress and strain components, while Tsai–Hill and Tsai–Wu criteria use polynomial expressions for stress interactions. Hashin criteria distinguish tensile and compressive modes dominated by the fibres or matrix, whereas Puck criteria evaluate fibre failure and inter-fibre failure using an action-plane approach. A Ye-type stress-separation criterion can additionally assess susceptibility to delamination initiation associated with through-thickness opening and interlaminar shear [22,23,24,25,26,27,28]. In the present study, these criteria were evaluated within a unified finite-element framework using the same three-dimensional stress history, enabling their predictions to be compared in terms of initiation stress, failure location, ply orientation, and governing mode.

Building on the combined-loading method evaluated previously [7], this study focuses on how end-tab geometry affects load transfer and failure initiation in ultra-thick CFRP compression specimens. Combined-loading compression tests on four tab configurations were used to compare load–displacement responses, compressive strengths, failure strains, and fracture patterns. Digital image correlation (DIC) was used to examine the evolution of representative free-edge strain localization, and scanning electron microscopy (SEM) was used to identify the associated microscale damage. A three-dimensional finite-element model of the specimen-tab assembly related the measured response to axial load redistribution, interlaminar stress concentrations, and first-ply failure initiation, and isolated the respective effects of taper length and tab thickness.

## 2. Materials and Methods

### 2.1. Materials and Specimen Configurations

The specimens were manufactured from GW800G/GS13 unidirectional carbon-fibre/epoxy prepreg supplied by Guangwei Composites (Weihai, Shandong, China). The prepreg had a nominal fibre areal mass of 190 ± 9 g/m^2^, a resin mass fraction of 34 ± 2%, and a cured ply thickness of approximately 0.187 mm. The laminate lay-up was [45/0/−45/90]_20s_, comprising 160 physical plies with a theoretical thickness of 29.92 mm. Staged debulking and closed-mould curing were used to control internal voids and thickness variation in the ultra-thick lay-up. Each specimen was 500 mm long and nominally 60 mm wide, with an untabbed gauge length of 150 mm. This geometry followed the combined-loading compression method for ultra-thick laminates previously evaluated experimentally by the authors [7].

G10/FR4 glass-fibre/epoxy tabs were bonded to the wide faces of the specimens using TS828 epoxy structural adhesive and cured for 24 h at room temperature under clamping. Before bonding, surface burrs were removed, and the bonding surfaces were cleaned with alcohol. The tapered configurations were denoted T20 and T15, and the abrupt-ended configurations were denoted A20 and A15. T20 and T15 both had a taper length of 25 mm; their taper angles were approximately 38.7° and 31.0°, and their nominal tab thicknesses were 20 and 15 mm, respectively. The A20 and A15 configurations employed abrupt-ended tabs with thicknesses of 20 and 15 mm, respectively. The prismatic section of each tab was 150 mm in length. Three specimens were tested for each configuration. Table 1 summarizes the tested configurations.

### 2.2. Compression Testing and Characterization

Compression tests were conducted using a horizontal servo-hydraulic system with a rated load capacity of 5000 kN (Figure 1). The combined-loading fixture transferred load through bearings at the specimen end faces and shear over the tab surfaces. High-stiffness platens, linear bearings, and a self-aligning mechanism were used to limit installation misalignment. After installation, each specimen was loaded to 100 kN to verify end-face contact, fixture alignment, and data acquisition. Loading was then continued at a displacement rate of 0.5 mm min^−1^ until the principal load drop. Nominal compressive stress was calculated from the axial load and the measured CFRP cross-sectional area according to Equation (1).

(1) $$\sigma_{c}=\frac{\left|F\right|}{Bt}$$

where *F* is the absolute axial load, and *B* and t are the measured width and thickness of the CFRP body, respectively. The maximum nominal stress before the principal load drop was defined as the experimental compressive strength.

Axial strain gauges were placed within the gauge section on the upper face, lower face, and one free edge of each specimen. One T20 specimen and one A20 specimen that provided complete and stable free-edge DIC fields throughout the pre-failure loading stage were selected to compare the two end geometries at a fixed nominal tab thickness of 20 mm. The x-axis was aligned with the loading direction and the z-axis with the thickness direction; the reported components were *ε_xx_*, *ε_xz_*, and *ε_zz_*. Samples from representative fracture regions were dried, mounted, sputter-coated with gold, and examined by SEM to identify fibre compression–shear fracture, matrix shear cracking, and interlaminar separation.

### 2.3. Three-Dimensional Finite-Element Model

A complete specimen-tab assembly was modelled in Abaqus/Standard 2024 (Figure 2). The CFRP and prismatic tab regions were discretized using C3D8R reduced-integration solid elements, and the tapered transition regions were discretized using C3D6 wedge elements. The in-plane element size in the gauge section was 2 mm, and the mesh was gradually coarsened along the longitudinal direction in the gripped regions. Mesh sensitivity was evaluated using in-plane element sizes of 1, 2, and 4 mm; the successive changes in the predicted Hashin first ply failure (FPF) stress were below 2%. An ideal tie constraint was imposed between each GFRP tab and the CFRP specimen to isolate the influence of tab geometry from adhesive damage and debonding. The tied interface isolates elastic geometry effects and does not represent adhesive deformation or damage. A symmetric nominal clamping pressure of 20 MPa was applied to the outer tab surfaces. The left reference point was fixed, while the transverse degrees of freedom of the right reference point were constrained and an axial displacement was prescribed. Loading was assumed to be ideally aligned, and geometric nonlinearity was disabled; the numerical interpretation is therefore confined to the pre-buckling response and local failure initiation. Three-dimensional stress fields at a nominal stress of approximately 300 MPa were used to compare load-transfer characteristics among the configurations. The orthotropic elastic properties, strengths, and Puck action-plane parameters used in the finite-element model are listed in Table 2.

The material parameters listed in Table 2 were obtained from tests conducted by the Testing and Evaluation Center for High-Performance Carbon Fiber, Beihang University, on unidirectional laminates manufactured from the same prepreg system. These parameters therefore correspond to the same underlying material system as the present specimens rather than to a different CFRP system reported in the literature. The GFRP tabs were modelled as equivalent orthotropic solids. The baseline T20 assembly contained 159,120 elements and 174,346 nodes. C3D8R elements used enhanced hourglass control, whereas C3D6 elements were restricted to the tapered transition. Element dimensions were approximately 2 mm in the gauge section and across the specimen width and 3 mm through the tab thickness and were gradually increased from 2 to 6 mm in the gripped regions. A single static step was used, in which the 20 MPa clamping pressure was maintained while the axial displacement at the guided loading reference point was increased smoothly to 5 mm. The imposed tab pressure and axial reference-point displacement provided an equivalent combined-loading boundary; actual grip–tab contact and friction were not modelled explicitly. Nominal stress was calculated by dividing the axial reaction force by the measured CFRP cross-sectional area. The FPF stress was obtained by linear interpolation between two successive output frames bracketing a failure index of unity.

### 2.4. Failure Indices and Parametric Cases

A universally accepted failure criterion capable of accurately characterizing the compressive failure of ultra-thick composite laminates has not yet been established. The pronounced three-dimensional stress state in these laminates may cause different criteria to produce distinct predictions of failure initiation. To evaluate this sensitivity, seven classical intralaminar failure criteria were considered: maximum strain, maximum stress, Tsai–Hill, two-dimensional and three-dimensional Tsai–Wu, three-dimensional Hashin, and three-dimensional Puck criteria [22,23,24,25,26,27]. The criteria were implemented through the user-defined output variable (UVARM) subroutine in Abaqus/Standard. At each material integration point, the subroutine retrieved the current stress and strain tensors and evaluated all failure indices in parallel along the same loading history.

The nominal compressive stress at which any failure index first reached unity was defined as the first-ply failure (FPF) stress, and the corresponding nominal strain was defined as the first-ply failure strain. The representative mathematical formulations of the selected criteria and their associated failure variables are summarized in Table 3. Among these criteria, Hashin 3D was subsequently used to examine geometry-induced transitions between fibre- and matrix-dominated failure initiation, whereas the remaining criteria were retained to assess the sensitivity of the predicted strength to the selected failure formulation. This first-ply failure event represents local criterion initiation in the linear-elastic model and is not interpreted as the experimental peak strength.

The fibre-tension, fibre-compression, matrix-tension, and matrix-compression components of the three-dimensional Hashin criterion [24,25] are expressed as Equations (2)–(5), respectively. Damage initiation was assumed to occur when the corresponding failure index reached unity.

(2) $$F_{ft}={\left(\frac{\sigma_{11}}{X_{T}}\right)}^{2}+{\left(\frac{\tau_{12}}{S_{12}}\right)}^{2}+{\left(\frac{\tau_{13}}{S_{13}}\right)}^{2}$$

(3) $$F_{fc}={\left(\frac{\sigma_{11}}{X_{C}}\right)}^{2}$$

(4) $$F_{mt}={\left(\frac{\sigma_{22}+\sigma_{33}}{Y_{T}}\right)}^{2}+\underset{ij=12,13,23}{\sum }{\left(\frac{\tau_{ij}}{S_{ij}}\right)}^{2}$$

(5) $$F_{mc}=\left[{\left(\frac{Y_{C}}{2S_{23}}\right)}^{2}-1\right]\frac{\sigma_{22}+\sigma_{33}}{Y_{C}}+{\left(\frac{\sigma_{22}+\sigma_{33}}{2S_{23}}\right)}^{2}+\frac{\tau_{23}^{2}-\sigma_{22}\sigma_{33}}{S_{23}^{2}}+\underset{ij=12,13}{\sum }{\left(\frac{\tau_{ij}}{S_{ij}}\right)}^{2}$$

Under transverse compression, the matrix-compression index includes contributions from transverse compression and all three shear components. Because the intralaminar criteria do not explicitly characterize delamination initiation at the free edge, a Ye-type stress-separation index was additionally used to assess susceptibility to delamination initiation associated with through-thickness opening and interlaminar shear [8,28]. Its governing equation is presented below:

(6) $$F_{Y}={\left(\frac{\max\left(\sigma_{33},0\right)}{Z_{T}}\right)}^{2}+{\left(\frac{\tau_{13}}{S_{13}}\right)}^{2}+{\left(\frac{\tau_{23}}{S_{23}}\right)}^{2}$$

This index separates the through-thickness opening term from the interlaminar shear terms and was used to locate regions susceptible to interlaminar failure initiation. The baseline geometry for the parametric study used a reinforcement tab with a taper length of 25 mm and a thickness of 20 mm. The taper-length cases were *L_s_* = 0, 12.5, 25, 37.5, and 50 mm, and the tab-thickness cases were *t_tab_* = 10, 15, 20, and 25 mm. Longitudinal paths were extracted along the mid-width line of the upper surface and, using symmetry, were plotted from the gauge centre to one gripped end. A through-thickness free-edge path was extracted at the gauge-tab transition. Results were averaged over every four adjacent physical plies to retain trends at the ply-block scale while suppressing individual-ply oscillations. Only the abrupt-ended and 25 mm taper geometries were tested experimentally; the remaining taper lengths were numerical sensitivity cases.

Equation (6) follows Ye’s stress-separation criterion, as subsequently applied to ultra-thick CFRP laminates. It represents delamination initiation as a quadratic interaction between the through-thickness normal stress and the two interlaminar shear stresses in material coordinates. The Macaulay operator ⟨*S*_33_⟩ = max (*S*_33_, 0) suppresses the normal-stress contribution under through-thickness compression, whereas *S*_13_ and *S*_23_ contribute through their squared normalized magnitudes. The formulation assumes a linear-elastic, pre-damage stress state and identifies initiation when the index reaches unity. Because it does not describe stiffness degradation, fracture-energy dissipation, or delamination growth, the Ye index is used only to compare local susceptibility to interlaminar damage initiation.

## 3. Results and Discussion

### 3.1. Mechanical Response and Fracture Morphology

During the initial loading stage, all four configurations entered a near-linear loading regime and exhibited an abrupt load drop at approximately 700–820 kN (Figure 3a), indicating rapid instability at the ultimate load. Although their pre-peak slopes were similar, the peak loads and the onset of the drop differed among the configurations. End-tab geometry therefore altered the load-carrying margin before catastrophic failure without substantially changing the overall elastic response of the gauge section.

T20 achieved the highest mean compressive strength of 439.57 MPa, whereas A20 reached 400.47 MPa. At a constant tab thickness of 20 mm, the 25 mm taper increased the strength by 39.10 MPa (9.76%) and the failure strain from 0.830% to 0.930%. These concurrent changes indicate that the taper delayed the point at which local failure became load-controlling, allowing further compression of the gauge section. For the 15 mm thick tabs, T15 and A15 reached strengths of 422.08 and 418.23 MPa, respectively.

The influence of tab thickness depended on the end geometry. Increasing the thickness from 15 to 20 mm raised the strength of the tapered configuration by 17.49 MPa but reduced that of the abrupt-ended configuration by 17.76 MPa. The measured modulus ranged from 47.8 to 52.2 GPa and showed no monotonic dependence on end geometry or thickness. A thicker tab can improve bearing support inside the grips, but it also increases the step change in sectional stiffness at an abrupt termination. The gradual thickness transition of the taper mitigates the latter effect, which explains why the 20 mm thickness produced a clear load-carrying benefit only in the tapered configuration.

All twelve specimens across the four configurations failed catastrophically within the unsupported gauge section; none showed end-crushing-dominated failure (Figure 4a–d). The fractures comprised longitudinal splitting of 0-degree fibre bundles, inclined compression–shear bands, matrix fragmentation in off-axis plies, and sublaminate separation. T20 and T15 showed extensive fibre brooming across the central gauge region, whereas A20 contained a more spatially concentrated crushed zone. A15 developed long axial splits and a pronounced wedge-shaped opening.

SEM examination provides further insight into the microscale damage processes responsible for the observed macroscopic fracture pattern (Figure 4e). The fracture surfaces exhibit multiple forms of local damage, including matrix-dominated cracking within the 90° plies, shear-induced matrix breakup and fibre exposure in the ±45° plies, and compressive fibre fracture accompanied by fibre rotation in the 0° plies. Evidence of local interlaminar debonding is also present. These features suggest that failure of the thick laminate does not arise from a single dominant mechanism. Instead, the final compression–shear fracture band develops through the combined evolution of matrix cracking and shear damage, fibre instability and breakage, and interlaminar separation [1,2,4]. The macroscopic fracture patterns and SEM observations indicate a coupled failure process rather than a single isolated mode. Matrix cracking and shear fragmentation in the 90° and ±45° plies can reduce transverse constraint around the load-bearing 0° bundles, while local fibre rotation and compression–shear fracture develop in the 0° plies. Sublaminate separation then links neighbouring intralaminar cracks.

### 3.2. Evolution of Free Edge Strain Fields

The tapered specimen maintained an approximately linear response throughout the intermediate loading stages, although the DIC-measured strain progressively exceeded the linear finite-element prediction after point C (Figure 5a). From points A to E, the $\varepsilon_{\mathrm{xx}}$ field remained broadly distributed across the gauge section while its compressive magnitude increased with loading. At point E, corresponding to a nominal stress of approximately 330 MPa, the displayed $\varepsilon_{\mathrm{xx}}$ range was −1.003% to −0.903%, whereas $\varepsilon_{\mathrm{xz}}$ and $\varepsilon_{\mathrm{zz}}$ remained within relatively narrow ranges of −0.084% to 0.071% and 0.310% to 0.413%, respectively. Immediately before macroscopic fracture at point F, however, the $\varepsilon_{\mathrm{xz}}$ range expanded markedly to −0.131% to 0.478%, while $\varepsilon_{\mathrm{zz}}$ increased to a range of −0.734% to 0.989%. The concurrent increase in magnitude and the emergence of both positive and negative values in these two components indicate the development of pronounced three-dimensional deformation involving coupled interlaminar shear, local bending, and through-thickness opening. The corresponding surface image further shows that this localization developed before fibre brooming and local delamination became evident within the gauge section.

The abrupt-ended specimen exhibited substantially earlier spatial localization (Figure 6). As the load increased, the measured stress–strain response progressively deviated from the linear finite-element prediction, while the damage image at point E revealed a localized fracture region near the tab termination. At point D, corresponding to approximately 335 MPa, the displayed ranges of $\varepsilon_{\mathrm{xx}}$, $\varepsilon_{\mathrm{xz}}$, and $\varepsilon_{\mathrm{zz}}$ were −0.958% to −0.703%, −0.135% to 0.162%, and 0.219% to 0.426%, respectively. Pronounced localized gradients were observed in all three strain components within approximately the same longitudinal region, indicating that the abrupt stiffness transition generated a coupled three-dimensional deformation field well before the final load drop.

A direct comparison can be made between points E and D, corresponding to similar nominal stresses of approximately 330 and 335 MPa for the tapered and abrupt-ended specimens, respectively. For the abrupt-ended specimen, the displayed strain ranges were 0.255% for $\varepsilon_{\mathrm{xx}}$, 0.297% for $\varepsilon_{\mathrm{xz}}$, and 0.207% for $\varepsilon_{\mathrm{zz}}$, compared with only 0.100%, 0.155%, and 0.103%, respectively, for the tapered specimen. Thus, before final fracture, the abrupt termination produced approximately twice the heterogeneity in the shear and through-thickness strain fields and about 2.6 times the heterogeneity in the axial strain field. Although substantially larger strain excursions were eventually observed in the tapered specimen at point F, these developed only during the terminal localization stage immediately preceding macroscopic fracture. These observations demonstrate that tapering does not eliminate the multiaxial strain localization associated with failure; rather, it delays its onset and suppresses its development over the intermediate and high-load stages.

### 3.3. Comparison of Failure-Initiation Criteria

As shown in Figure 7, the intralaminar failure criteria produced different predictions of damage initiation for a given configuration and loading path, demonstrating the sensitivity of their failure envelopes to the treatment of three-dimensional stress components and their interactions. In terms of strength prediction (Figure 7a), Puck 3D, the maximum stress criterion, and Tsai–Hill produced the smallest numerical differences from the measured ultimate strengths. Their mean absolute percentage differences relative to the measured strengths of the four configurations were 3.3%, 3.5%, and 3.5%, respectively (Figure 7b), indicating that these criteria provide a reasonable representation of the overall strength level of the present specimens. Hashin 3D was more sensitive to local matrix compression in configurations with abrupt geometric transitions and therefore predicted earlier damage initiation in some cases. Its principal advantage, however, is its ability to distinguish between fibre-compression and matrix-compression failure modes. By contrast, Tsai–Wu 2D and Tsai–Wu 3D generally yielded higher initiation stresses, suggesting a stronger influence of their polynomial interaction terms on the predicted failure envelope under the present three-dimensional stress state. Because FPF denotes local initiation rather than macroscopic collapse, these differences describe numerical proximity to the experimental peak and are not treated as direct validation of ultimate-strength prediction.

The predicted failure strains exhibited a grouping similar to that observed for the strength predictions (Figure 7c). Prior to damage initiation, the specimens remained approximately linear elastic; consequently, the different criteria identified failure at different thresholds along essentially the same stress–strain loading path. Differences in the predicted initiation strength were therefore reflected directly in the corresponding initiation strains. It should be noted, however, that the experimental failure strain and the first-ply failure strain obtained from UVARM do not represent exactly the same physical event. The numerical value corresponds to the onset of failure at the first critical material point, whereas the experimentally measured failure strain is associated with the subsequent macroscopic loss of load-carrying capacity and thus includes the interval over which initially localized damage develops and propagates before the principal load drop. This distinction explains why the experimental failure strains were generally higher than the UVARM predictions.

The configuration-wise prediction errors (Figure 7d) further show that the performance of each criterion depends not only on its overall mean error but also on changes in specimen geometry and the associated local stress state. Accordingly, the suitability of a failure criterion cannot be established solely from a single global error metric; its ability to identify physically relevant failure modes and geometric effects must also be considered. On this basis, Puck 3D, the maximum stress criterion, and Tsai–Hill were used to evaluate the overall strength level of the different configurations, whereas Hashin 3D was employed to track transitions between fibre-compression and matrix-compression failure induced by geometric variations. Independently of these intralaminar criteria, the Ye index was used to characterize the risk of interlaminar damage initiation near the free edge.

### 3.4. Effect of Taper Length on Load Transfer and Failure Initiation

Taper length was the dominant geometric parameter governing load transfer at the tab termination. The near-abrupt configuration developed a narrow $S_{11}$ gradient, an intense $S_{13}$ shear band, and a region of $S_{33}$ compression at the termination plane. As $L_{s}$ increased, the high-gradient region extended along the loading direction, and the peak magnitudes decreased (Figure 8). This response is consistent with shear-lag load transfer: distributing the same increment in axial force over a longer stiffness-transition region reduces the shear force transferred per unit length by the interface and neighbouring plies.

The path results further quantify the influence of the transition length (Figure 9). As $L_{s}$ increased from 0 to 50 mm, the peak normalized longitudinal compressive stress, $-S_{11}/\sigma_{\mathrm{nom}}$, decreased from 1.521 to 1.234, while the peak $\left|S_{13}\right|$ decreased from 48.01 to 18.36 MPa. Meanwhile, the minimum $S_{33}$ became less compressive, increasing from −60.19 to approximately −20.08 MPa. Correspondingly, the Hashin-predicted first-ply failure stress increased from 326.53 to 457.72 MPa. Matrix compression governed the initial failure for $L_{s}=0$ and 12.5 mm, whereas fibre compression became dominant for $L_{s}>25$ mm. These results indicate that increasing the transition length progressively alleviates the local transverse compression and shear concentrations near the termination. Once these local stress concentrations are sufficiently suppressed, failure is no longer controlled by matrix-dominated damage at the termination but instead shifts to fibre compression in the load-bearing 0° plies.

The four-ply-averaged responses at the free edge on the gauge side of the tab termination show how end geometry altered through-thickness load distribution (Figure 10). The surface-ply values of $-S_{11}/\sigma_{nom}$ reached 1.211 for T20 and 1.165 for T15, indicating progressive transfer of axial l a surface-ply concentration of through-thickness stress, whereas the taper allowed the surface axial stress to develop progressively and reduced the normal-stress concentration.

The peak |S_13_| ranged from 18.33 to 22.23 MPa across the four configurations. Because $S_{33}$ remained compressive along the sampled path, the opening term max($S_{33}$, 0) in the Ye index was inactive at these locations; the higher Ye indices of A20 and A15 were therefore governed primarily by the interlaminar shear terms. At the measured peak-load levels, the calculated Ye indices were 0.53 and 0.42 for A20 and A15, compared with 0.21 and 0.12 for T20 and T15. Because stiffness was not degraded after FPF, these peak-load values are comparative elastic risk indices rather than post-damage stress states. Their relative increase is qualitatively consistent with the earlier localization observed in the representative A20 specimen and with the delamination found in the final fracture region.

### 3.5. Effect of Tab Thickness and Interaction with End Geometry

At approximately 300 MPa, increasing $t_{tab}$ from 10 to 25 mm intensified the $S_{11}$ gradient at the tapered termination and expanded the elevated $S_{13}$ band and the $S_{33}$ compression region (Figure 11). A thicker tab increased the axial stiffness of the combined section in the gripped region. The same far-field load therefore had to be redistributed into the untabbed CFRP section through greater interlaminar shear and stronger through-thickness constraint.

The path results further quantify this redistribution (Figure 12). As $t_{tab}$ increased from 10 to 25 mm, the peak $-S_{11}/\sigma_{nom}$ increased from 1.194 to 1.405, while the peak $\left|S_{13}\right|$ increased from 16.14 to 30.98 MPa, and the minimum $S_{33}$ decreased from −20.24 to −30.85 MPa. The corresponding Hashin first-ply failure stress decreased from 476.19 to 421.79 MPa. At a fixed taper length of 25 mm, increasing tab thickness alone intensified the stiffness mismatch at the termination and reduced the local failure margin. Relative to the 10 mm tab, the 25 mm tab increased the peak normalized *S*_11_ by 17.7%, increased the peak *S*_13_ by 91.9%, and increased the magnitude of the compressive *S*_33_ minimum by 52.4%; the predicted Hashin first-ply failure stress decreased by 11.4%. These changes arise because the axial stiffness per unit width of the tabbed section increases with tab thickness. A larger fraction of the applied force is carried through the tab in the gripped region and must be transferred back into the CFRP over the finite termination length, increasing the required interlaminar shear and the accompanying through-thickness constraint. Tab thickness is therefore not an independent strengthening variable: its effect is governed by how rapidly the associated stiffness increment is terminated.

The experimental thickness trend depended on end geometry: T20 was 17.49 MPa stronger than T15, whereas A20 was 17.76 MPa weaker than A15. This contrast differs from the numerical trend at *Ls* = 25 mm, for which increasing tab thickness intensified the local *S*_13_ and *S*_33_ concentrations and reduced the Hashin FPF stress. The difference may reflect mechanisms not represented by the idealized elastic model, including grip contact and adhesive compliance, and is therefore not used to infer a general beneficial effect of tab thickness. For the tapered configurations, Hashin, maximum stress, Tsai–Hill, and Puck 3D predictions fell within a relatively narrow interval, indicating that their initiation estimates were governed mainly by the axial load-carrying component after the local three-dimensional stress concentration had been reduced. For the abrupt-ended configurations, Hashin and maximum strain responded earlier to the local combination of matrix compression and shear, whereas maximum stress, Tsai–Hill, and Puck 3D remained close to the experimental strength. End-transition geometry therefore affected both the load-carrying level and the sensitivity of the criteria to the local three-dimensional stress state. Accordingly, the experimental and numerical thickness trends are reported as configuration-specific observations rather than as evidence that increasing tab thickness is generally beneficial.

## 4. Conclusions

Combined-loading compression tests, surface-strain and fractographic characterization, and multi-criterion three-dimensional finite-element analyses were used to determine how end-tab geometry governs load transfer and failure initiation in ultra-thick CFRP specimens. The following conclusions were drawn:

1. For the 20 mm thick tabs tested in triplicate, a 25 mm taper increased the mean compressive strength by 9.76% and increased the mean failure strain. For the specimens with 15 mm thick tabs, only a small strength difference was observed between the tapered and abrupt-ended configurations. The measured thickness effect therefore depended on the stiffness transition at the tab termination.
2. At comparable nominal stresses of approximately 330–335 MPa, the representative abrupt-ended specimen exhibited approximately twice the min-to-max strain ranges for the shear and through-thickness components and 2.6 times the axial-strain span of the representative tapered specimen. The tapered specimen developed severe coupled localization only immediately before fracture, indicating delayed development of the concentrated free-edge deformation field.
3. As $L_{s}$ increased from the near-abrupt condition to 50 mm, the peak $\left|S_{13}\right|$ decreased from approximately 48.0 to 18.4 MPa, the minimum *S*_33_ increased from approximately −60.2 to −20.1 MPa, and the governing Hashin mode changed from matrix compression to fibre compression. Taper length was the principal geometric parameter controlling load transfer and the predicted initiation mode at the termination.
4. Puck 3D, maximum stress, and Tsai–Hill produced initiation stresses numerically closest to the measured ultimate-strength levels, whereas Hashin 3D identified the geometry-induced transition between matrix- and fibre-controlled initiation. The higher Ye indices of the abrupt-ended configurations were qualitatively consistent with their greater interlaminar stress concentration and the delamination observed after failure.

Among the tested configurations, the 25 mm taper with a 20 mm thick tab produced the highest measured strength; this configuration is not claimed as a global optimum. Application to other material systems, specimen thicknesses, adhesives, or clamping conditions requires reassessment of the free-edge stress state and interfacial capacity.

## Figures and Tables

**Figure 1:**
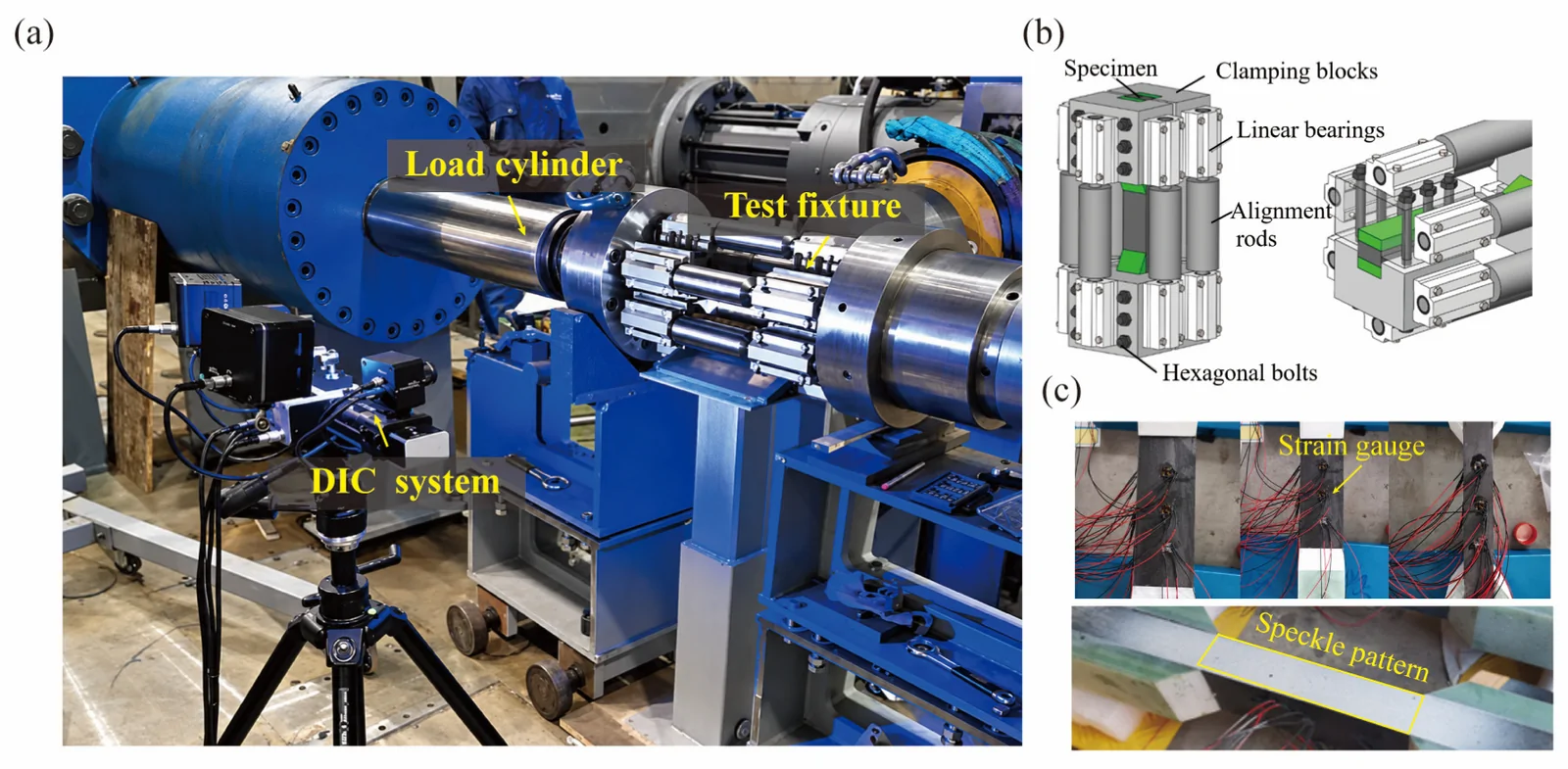
Compression test system (**a**), combined-loading fixture (**b**), and strain-gauge and DIC arrangements (**c**).

**Figure 2:**
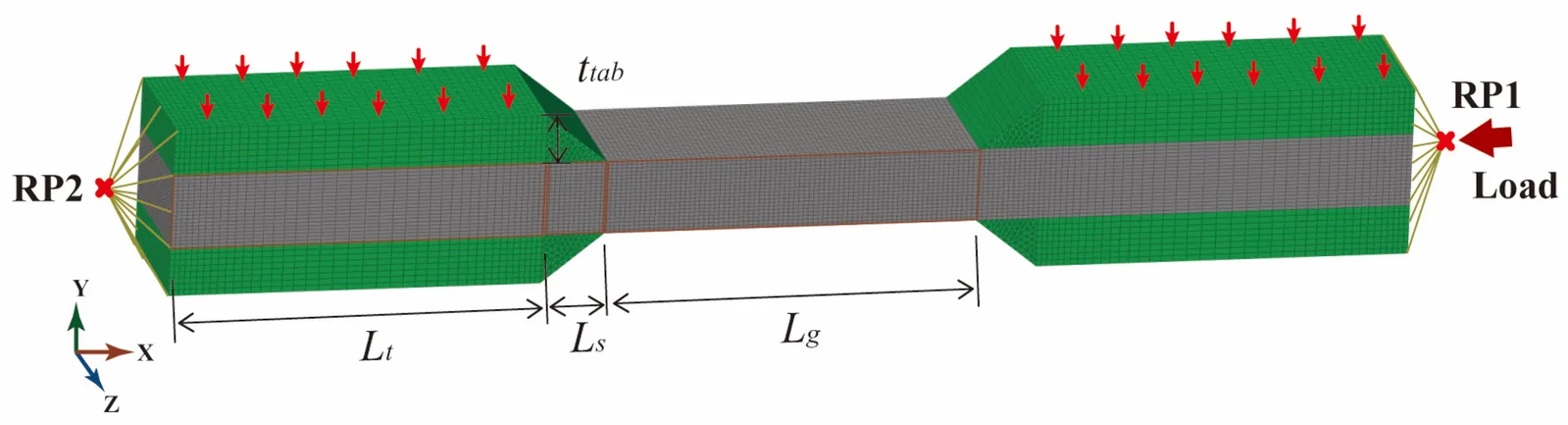
Three-dimensional finite-element model of the complete specimen-tab assembly, including the mesh and boundary conditions. The arrows indicate the applied axial loading and clamping-pressure directions.

**Figure 3:**
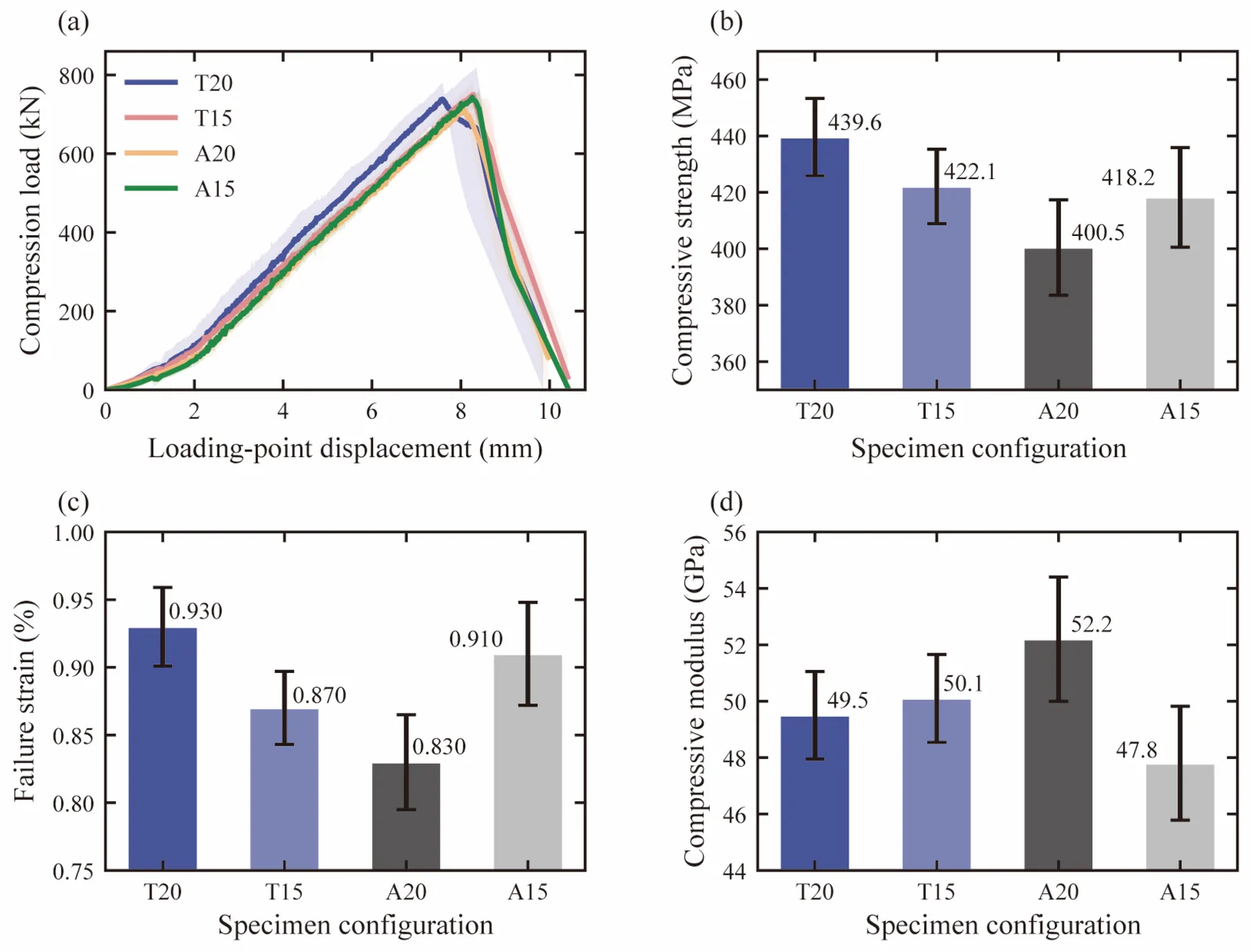
Mechanical responses of specimens with the four end-tab configurations: (**a**) load–displacement responses; (**b**) compressive strength; (**c**) failure strain; and (**d**) compressive modulus.

**Figure 4:**
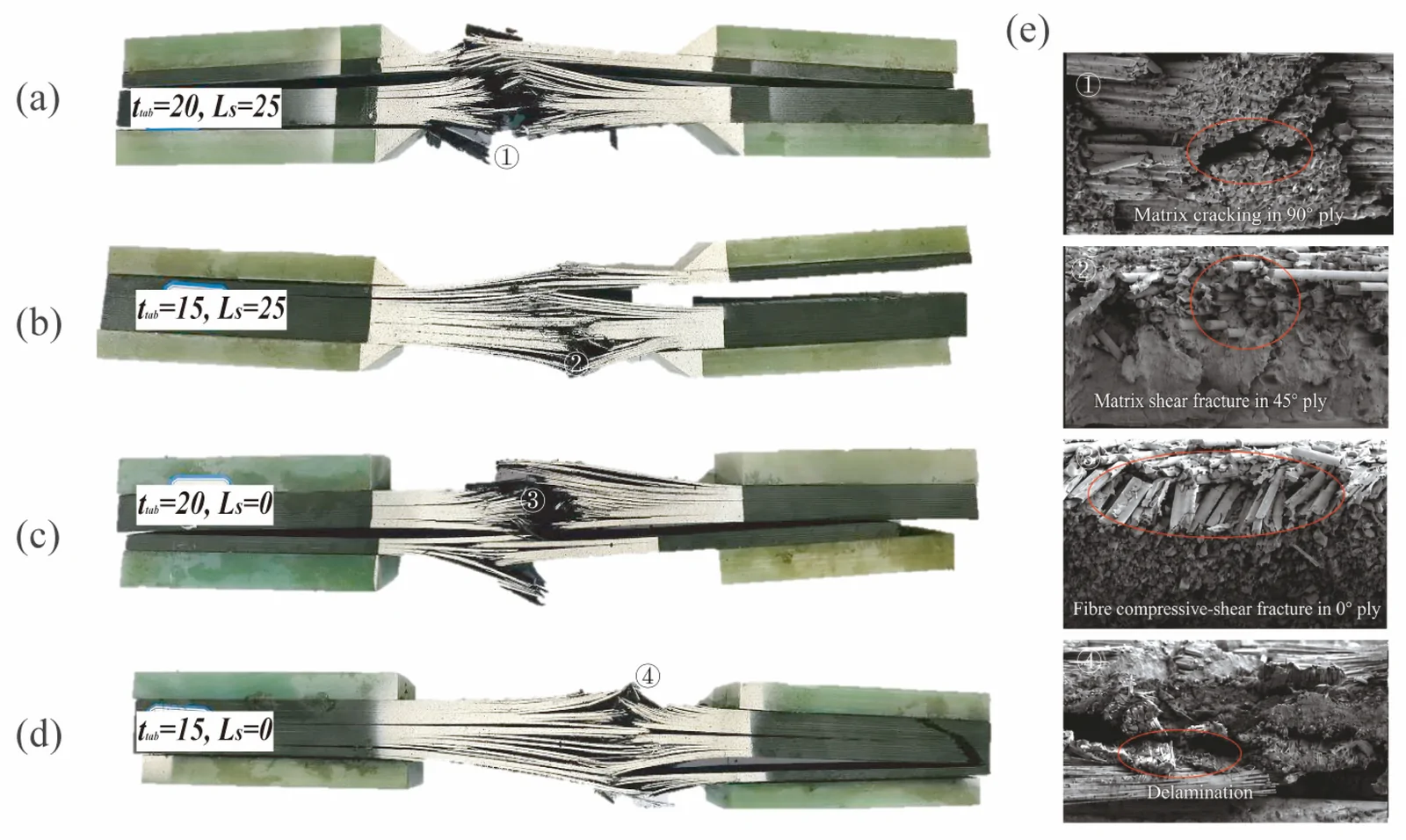
Macroscopic fracture patterns and corresponding SEM observations: (**a**) T20; (**b**) T15; (**c**) A20; (**d**) A15; and (**e**) microscale features at the numbered locations.

**Figure 5:**
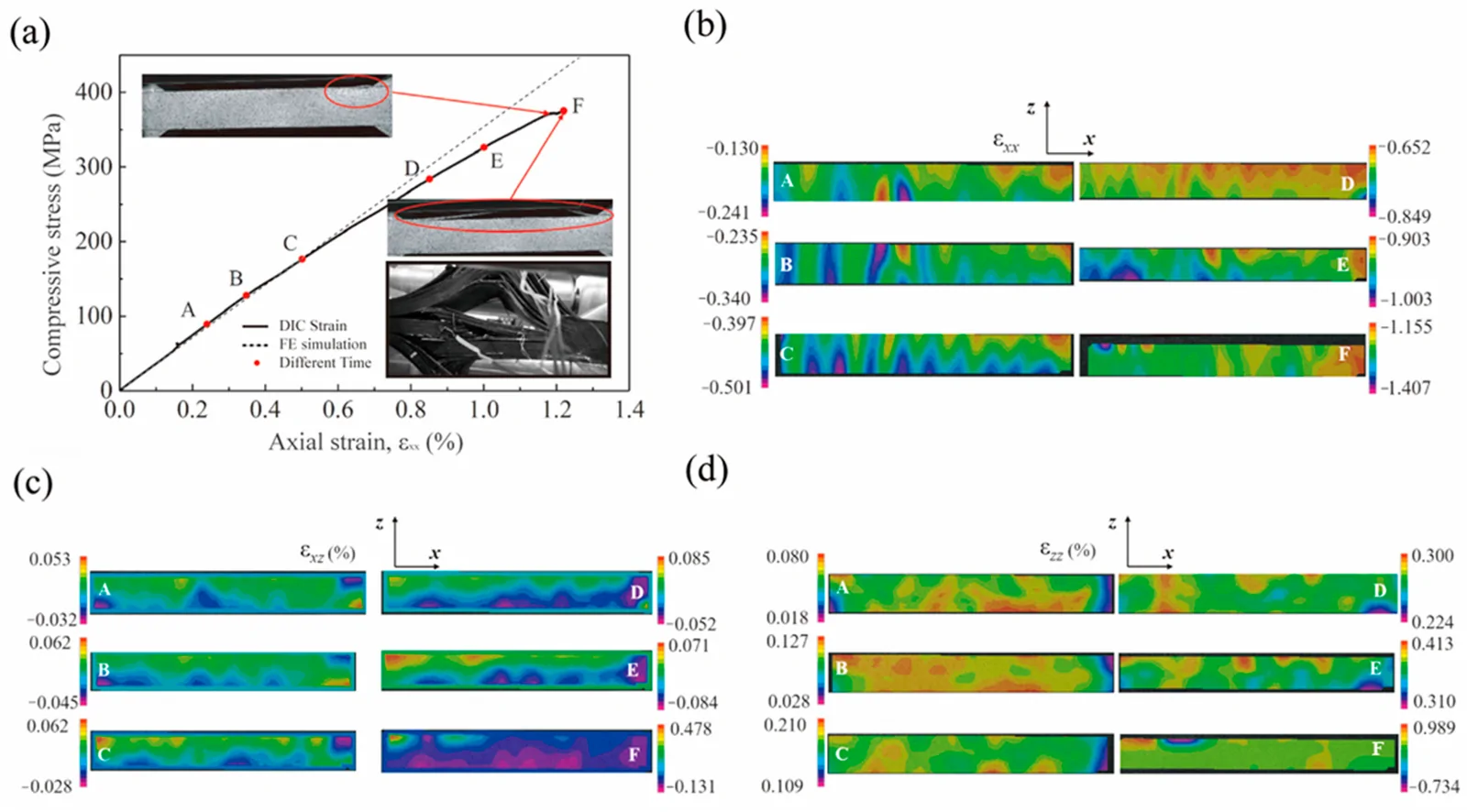
DIC response of the representative tapered specimen: (**a**) measured stress–strain response, linear finite-element response, and selected instants A–F; (**b**) $\varepsilon_{xx}$; (**c**) $\varepsilon_{xz}$; and (**d**) $\varepsilon_{zz}$. Letters A–F in subfigures (**b**–**d**) correspond to the loading stages marked on the stress–strain curve in (**a**).

**Figure 6:**
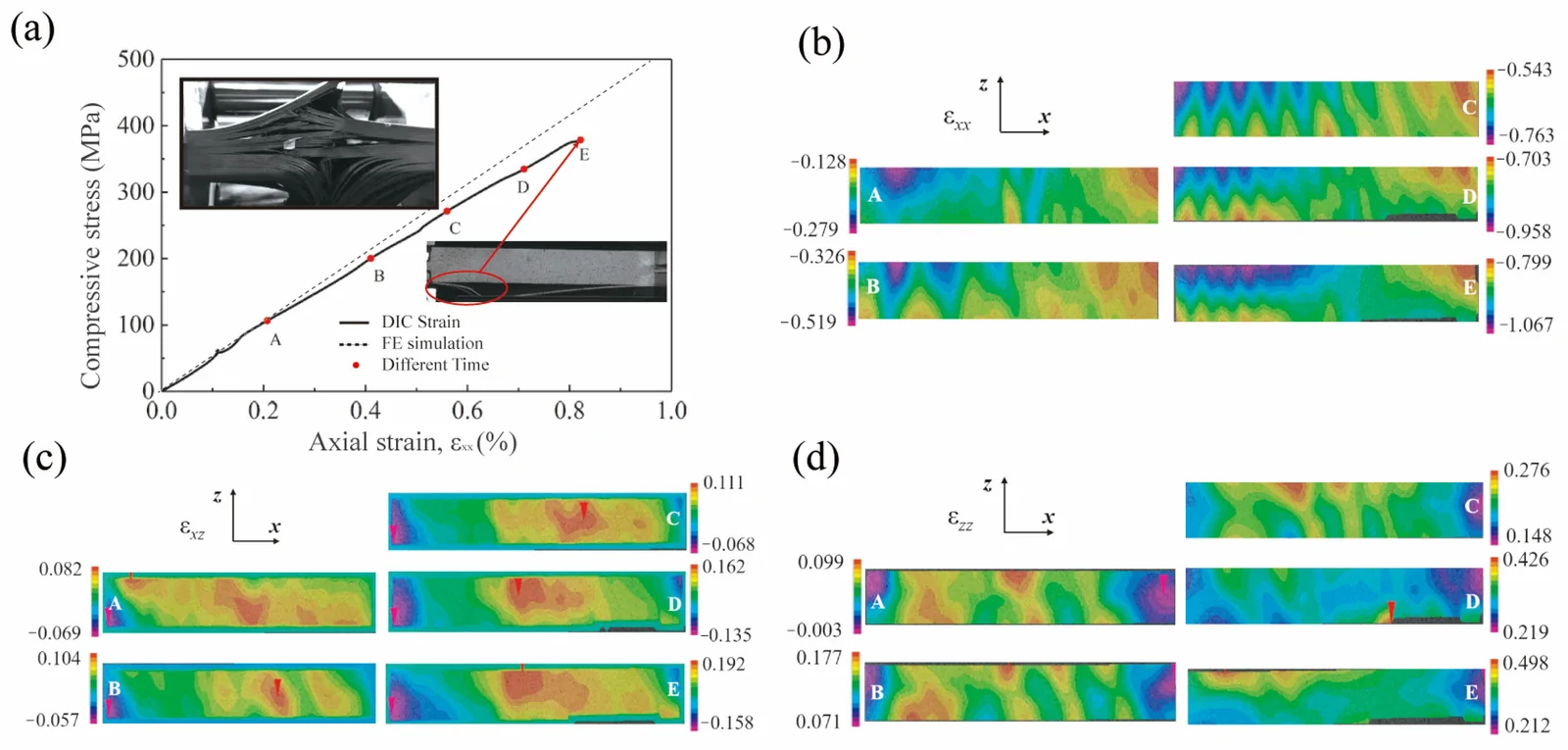
DIC response of the representative abrupt-ended specimen: (**a**) measured stress–strain response, linear finite-element response, and selected instants A–E; (**b**) $\varepsilon_{xx}$; (**c**) $\varepsilon_{xz}$; and (**d**) $\varepsilon_{zz}$. Letters A–E in subfigures (**b**–**d**) correspond to the loading stages marked on the stress–strain curve in (**a**).

**Figure 7:**
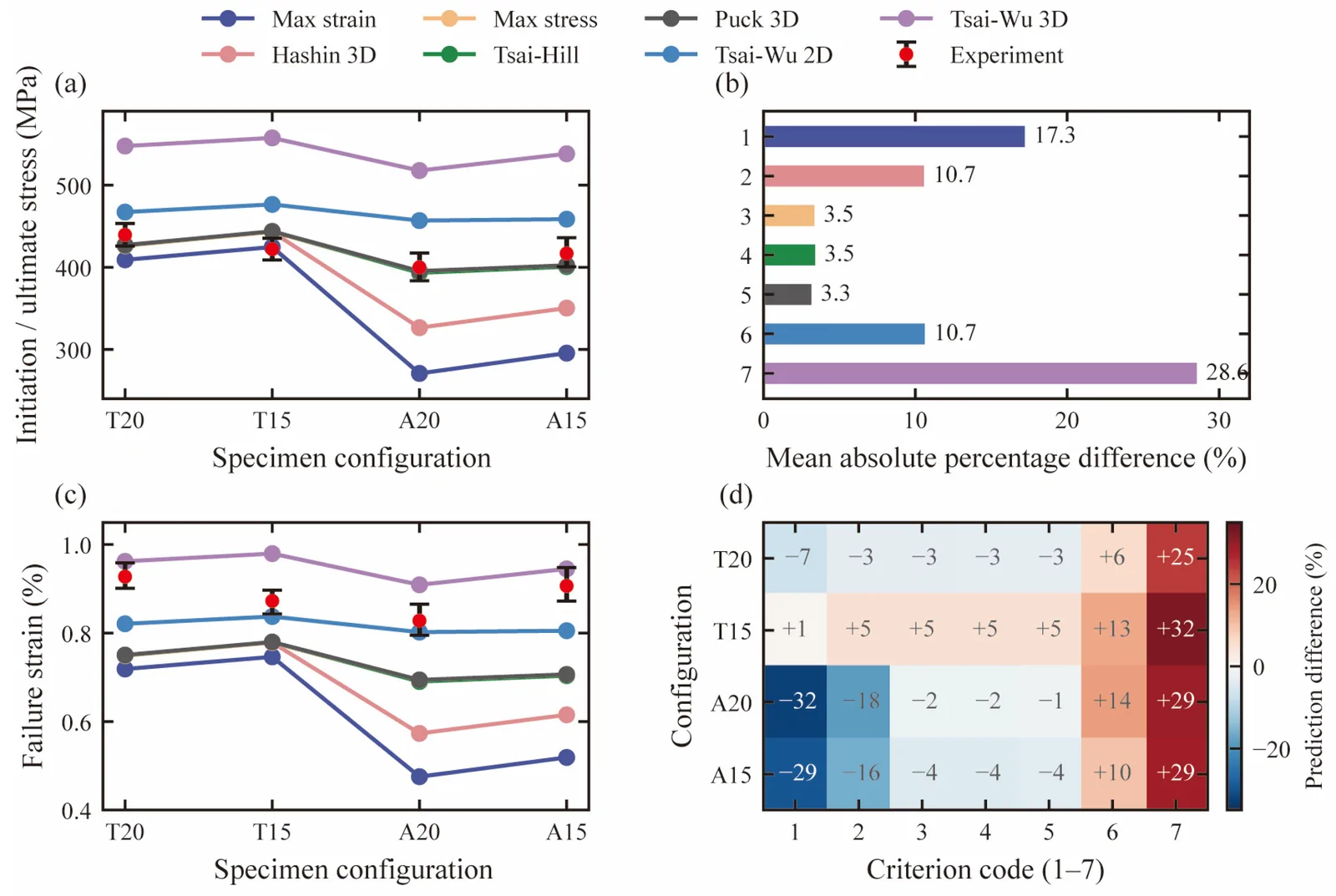
Assessment of the seven intralaminar failure criteria: (**a**) predicted initiation stresses and experimental strengths; (**b**) mean absolute percentage difference; (**c**) predicted and experimental failure strains; and (**d**) difference/deviation relative to the experimental peak strength. Criterion codes 1–7 denote maximum strain, maximum stress, Hashin 3D, Puck 3D, Tsai–Hill, Tsai–Wu 2D, and Tsai–Wu 3D, respectively.

**Figure 8:**
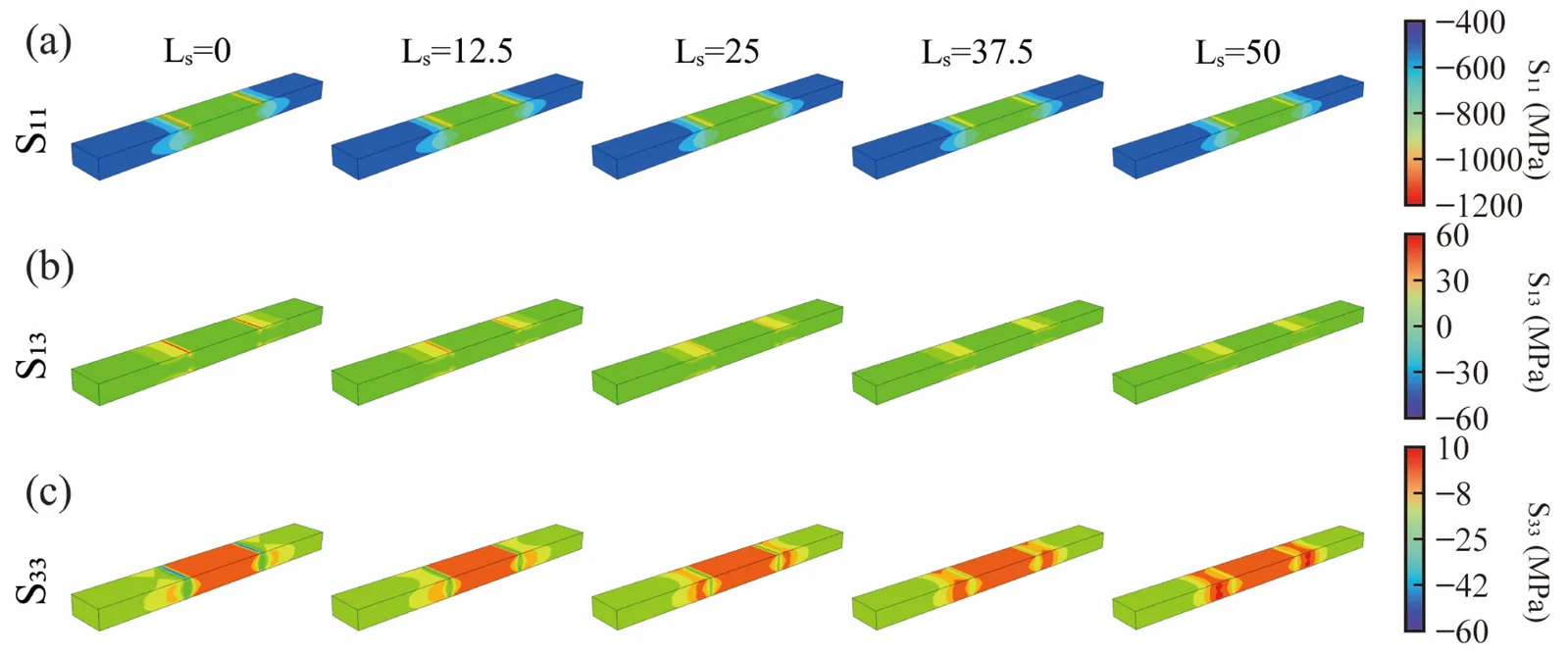
Three-dimensional stress fields in specimens with different taper lengths at approximately 300 MPa: (**a**) $S_{11}$; (**b**) $S_{13}$; and (**c**) $S_{33}$.

**Figure 9:**
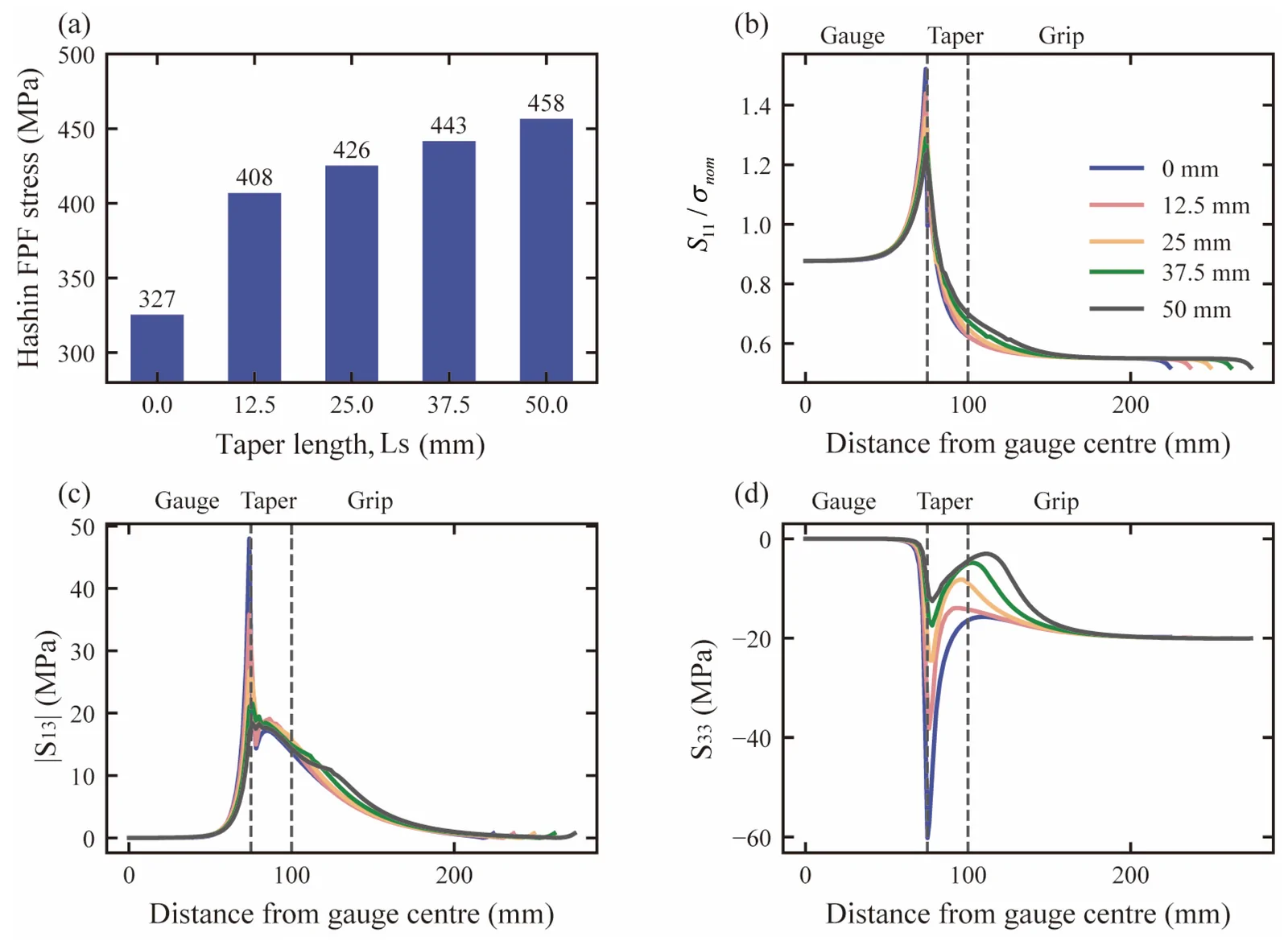
Effects of taper length on (**a**) the Hashin 3D first-ply failure stress; (**b**) $-S_{11}/\sigma_{nom}$; (**c**) $\left|S_{13}\right|$; and (**d**) $S_{33}$. The path extends from the gauge centre to one gripped end.

**Figure 10:**
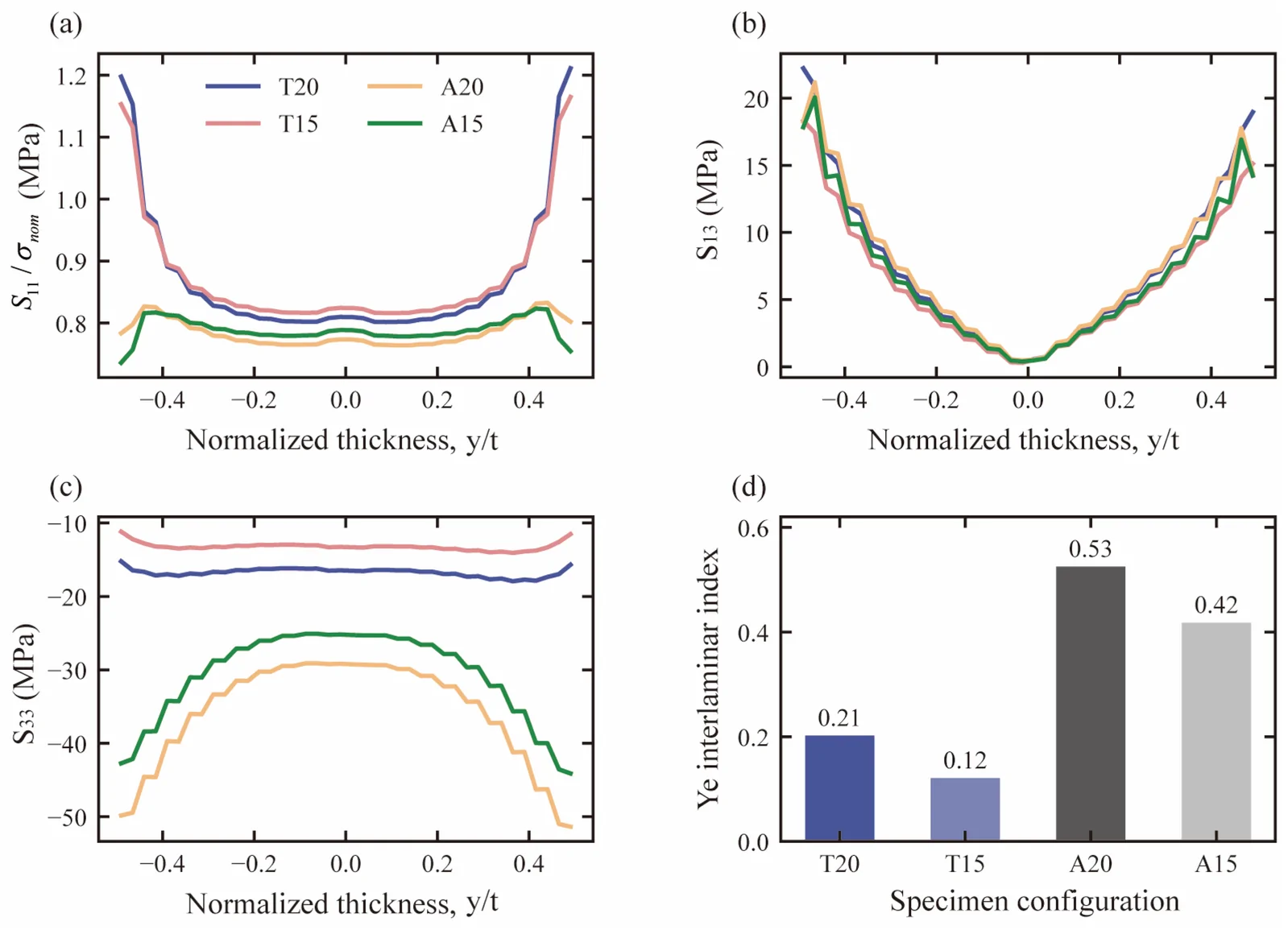
Four-ply-averaged responses at the free edge on the gauge side of the tab termination: (**a**) $-S_{11}/\sigma_{nom}$; (**b**) $\left|S_{13}\right|$; (**c**) $S_{33}$; and (**d**) the Ye interlaminar index at the experimental peak load.

**Figure 11:**
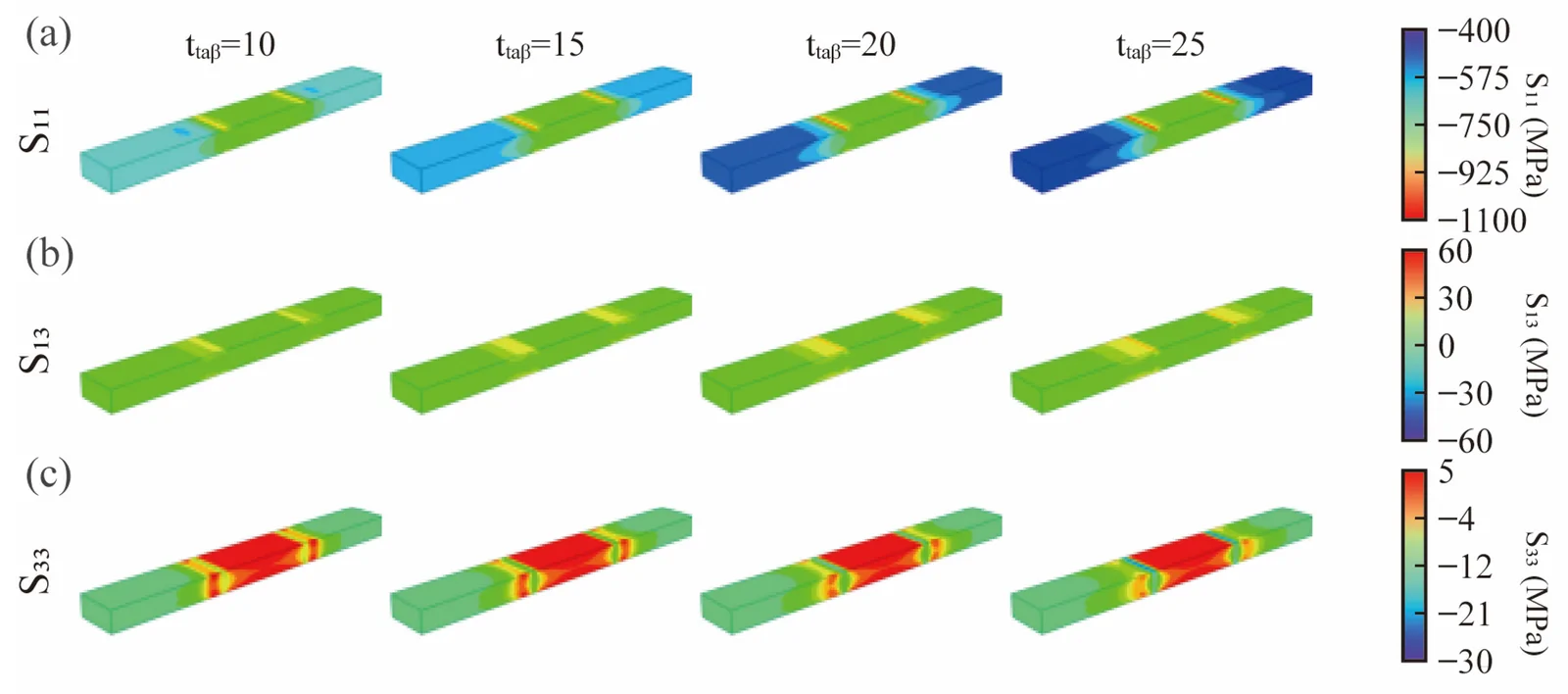
Three-dimensional stress fields in specimens with different tab thicknesses at approximately 300 MPa: (**a**) $S_{11}$; (**b**) $S_{13}$; and (**c**) $S_{33}$.

**Figure 12:**
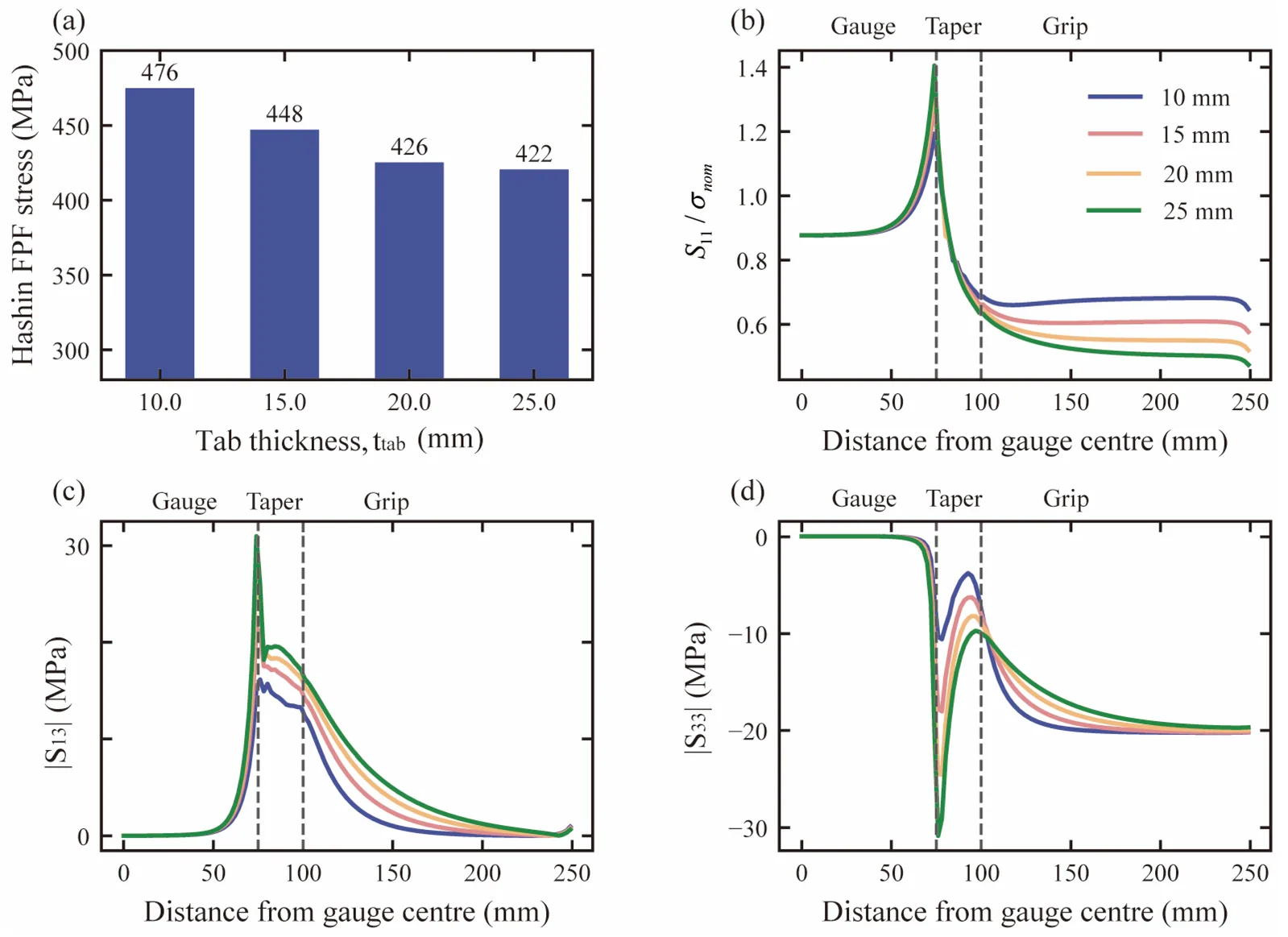
Effects of tab thickness on (**a**) the Hashin 3D first-ply failure stress; (**b**) $-S_{11}/\sigma_{nom}$; (**c**) $\left|S_{13}\right|$; and (**d**) $S_{33}$.

**Table 1:** Geometric dimensions of specimens with different end-tab configurations.

Configuration	End Geometry	$L_{s}$ (mm)	$t_{tab}$ (mm)	Specimen Dimension *L* × *B* × *T* (mm)
T20	Tapered	25	20	500 × 60 × 30
T15	Tapered	25	15	500 × 60 × 30
A20	Abrupt	0	20	500 × 60 × 30
A15	Abrupt	0	15	500 × 60 × 30

**Table 2:** CFRP elastic properties, strengths, and Puck action-plane parameters.

Parameter Category	Parameters and Values
Elastic properties	$E_{1}=$ 147,000 MPa; $E_{2}=E_{3}=9320$ MPa; $G_{12}=G_{13}=4530$ MPa; $G_{23}=3900$ MPa; $\nu_{12}=\nu_{13}=\nu_{23}=0.30$
Strength parameters	$X_{T}=2520$ MPa; $X_{C}=1528$ MPa; $Y_{T}=37.5$ MPa; $Y_{C}=160$ MPa; $Z_{T}=29.8$ MPa; $Z_{C}=160$ MPa; $S_{12}=S_{13}=S_{23}=122$ MPa
Puck parameters	θ=±53°; $p_{c}=0.25;$ $p_{tp}=0.30;$ $p_{cp}=0.22;$ $\nu_{f}=0.20;$ $m_{\sigma f}=1.10$

**Table 3:** Intralaminar failure criteria implemented through UVARM.

Criterion	Representative Mathematical Form
Maximum stress	$F_{MS}=\max\left|\sigma_{i}/R_{i}\right|$
Maximum strain	$F_{ME}=\max\left|\varepsilon_{i}/\varepsilon_{i}^{u}\right|$
Tsai–Hill	$F_{TH}={\left(\sigma_{11}/R_{1}\right)}^{2}-\sigma_{11}\sigma_{22}/R_{1}^{2}+{\left(\sigma_{22}/R_{2}\right)}^{2}+{\left(\tau_{12}/S_{12}\right)}^{2}$
Tsai–Wu 2D/3D	$F_{TW}=F_{i}\sigma_{i}+F_{ij}\sigma_{i}\sigma_{j}$
Hashin 3D	$F_{H}=\max\left(F_{ft},F_{fc},F_{mt},F_{mc}\right)$
Puck 3D	$F_{P}=\max\left(F_{FF},F_{IFF}^{+53{}^{\circ}},F_{IFF}^{-53{}^{\circ}}\right)$

## Data Availability

The original contributions presented in this study are included in the article. Further inquiries can be directed to the corresponding author.
